# Association between BMI z-score and body composition indexes with blood pressure and grip strength in school-age children: a cross-sectional study

**DOI:** 10.1038/s41598-024-55875-z

**Published:** 2024-03-05

**Authors:** Paola Vanessa Miranda-Alatriste, Eloisa Colin-Ramirez, Patricia Inda Icaza, Xóchitl Ponce-Martínez, Anette Shor Mochón, Natalia Martinsanchez Vázquez, Berenice García-Arreola, María de los Ángeles Espinosa-Cuevas

**Affiliations:** 1 Instituto Nacional de Ciencias Médicas y Nutrición Salvador Zubirán, 14080 Mexico City, Mexico; 2 School of Sport Sciences, Universidad Anáhuac México, Av. Universidad Anáhuac 46, Lomas Anáhuac, 52786 Huixquilucan, Mexico; 3 Universidad Anáhuac México, 52786 Huixquilucan, Mexico; 4 Universidad Iberoamericana Puebla, 72810 San Andrés Cholula, Mexico

**Keywords:** Elevated blood pressure, Adiposity, Lean mass, Cardiovascular risk, Childhood obesity, Cardiology, Risk factors

## Abstract

Childhood obesity is linked to diverse health outcomes, including elevated blood pressure (EBP). Emerging evidence showed that excess fat mass (FM) may have a deleterious impact on blood pressure even in normal-weight children. The primary objective of this study was to assess the association between body weight status by BMI z-score and body composition parameters by conventional bioelectrical impedance analysis (BIA) and bioelectrical impedance vector analysis (BIVA). Also, we aimed to explore the performance of BMI z-score, %FM, and FM index (FMI) in discriminating EBP in a sample of school-age Mexican children. Children were classified as having normal weight, overweight or obesity according to WHO criteria for BMI z-score. FMI was considered high when above 75th percentile, and fat free mass index (FFMI) was considered low when below 25th percentile of the reference population. Body composition was also classified according to the BIVA method and EBP was determined when systolic and/or diastolic blood pressure ≥ 90th percentile. BMI z-score groups were compared by Student´s t-test or the Mann–Whitney U test, or by the chi-square test or Fisher exact test. Receiving operating characteristic (ROC) analysis was performed. 61 children were included (52.5% boys, median age 9.8 (25th, 75th percentiles: 8.5, 11.0)) years. High FMI was observed in 32.3% of children with normal weight. Low FFMI was present in 93.5% of children with normal weight and 53.3% of those with overweight/obesity. According to BIVA, 58.1% and 43.3% of children with normal weight and overweight/obesity were classified as having cachexia. All the three adiposity indicators showed significant areas under the ROC curve (AURC) greater than 0.775 for EBP, with the largest one displayed for FM% (0.794). Hight FMI and low FFMI are common in children with normal weight. Identifying deficiency of FFM might be limited by using solely BMI indicators. Cachexia by BIVA was present in a high proportion of children with either normal weight or overweight/obesity. Both BMI z-score and FM (% and FMI) performed well at discriminating EBP, with a numerically greater AURC observed for FM%. Body composition in pediatric population is relevant for identifying body composition abnormalities at early age.

## Introduction

Childhood obesity is a relevant public health issue globally and continues to increase particularly in low- and middle-income countries, where it coexists with diverse forms of undernutrition^1^. In Mexico, current combined overweight and obesity rate in school-age children is estimated to be 37%^2^. There is strong evidence linking childhood obesity to diverse health outcomes, including elevated blood pressure (EBP)^3,4^. A large analysis of data from European children and adolescent reveled that the prevalence of primary hypertension ranged from 5% in normal weight children up to 39% in children with severe obesity^5^. However, recent data suggest that excess fat mass (FM) may have a delirious impact on blood pressure levels even in normal-weight children^6^, pointing out the challenge of health risk stratification based on BMI classification in children^7^, since it does not allow to distinguish between different body compartments.

On the other hand, handgrip strength test is widely used in the pediatric population as a good proxy of whole-body muscle strength due to its strong correlation with whole-body muscle strength in children and adolescents; clinically, it is also considered as an indicator of muscle functionality^8^. Low muscle mass and strength have also been linked to adverse health outcomes in childhood^8,9^; in fact, a large cross-sectional study in children and adolescents reported an adjusted decreased risk of having high systolic blood pressure associated with increased lean soft tissue (LST) by dual x-ray absorptiometry (DXA)^10^. In addition, it has been suggested that the co-existence of obesity and reduced muscle mass, which is known as sarcopenic obesity, may imply more adverse effects that obesity alone. However, this and other body composition phenotypes and their health implications have been poorly studied in children; in part, due to the lack of consensus for its definition and diagnostic methods^11^, requiring population-specific normative data. Bioelectrical impedance analysis (BIA) is a non-invasive and convenient method to assess the body composition in pediatric population, and has been demonstrated to be accurate relative to DXA^12^. Besides conventional BIA, which uses prediction equations to estimate body compartments, the bioelectrical impedance vector analysis (BIVA) has been proposed as an alternative method to assess body composition of children without making any of the assumption required for conventional BIA^13^.

Taking all together, it is relevant to generate evidence regarding the utility of the non-invasive BIA method to identify body composition alterations of children at different body weight levels and its association with health outcomes.

The primary objective of this study was to assess the association between body weight status by BMI z-score and body composition parameters by conventional BIA and BIVA. Secondarily, we aimed to explore the association between BIVA and hand-grip strength, as well as to assess the performance of BMI z-score and body composition parameters in discriminating EBP in a sample of school-age Mexican children.

## Methods

### Study sample and recruitment

This cross-sectional study is a pilot of a larger study aimed at evaluating the association between different phenotypes of body composition with cardio-metabolic risk markers and gut microbiota profiles in Mexican school-age children. For this pilot, a convenience sample of 61 healthy children were consecutively recruited from 3 public elementary schools located in Mexico City and conurbation area from May to September 2023. Children were also recruited from the community by a word-of-mouth strategy during the same period. Children of any sex were included if they were 8–12 years old and excluded if they had a chronic disease such as hypertension, cancer, diabetes, heart, renal or liver disease, pacemaker or limb amputation.

Participating public schools’ authorities allowed the research team members to meet with parents at school during an informative meeting to invite them to take place in the study. Parents who were interested were provided with further detailed information and eligibility was confirmed. Participants’ parents recruited from the community contacted the research team members for detailed information and eligibility confirmation.

The study protocol was approved by the Anahuac University Research and Bioethics Committees under number 202302. Research was performed in accordance with the Declaration of Helsinki. Parents or guardians provided written informed consent and children provided assent to be involved in the study.

### Assessments

Study visits were conducted at school in a private room assigned for this purpose. Children recruited from the community were evaluated in a consultation office enabled for this research purpose. Anthropometric, hand-grip strength, body composition, and blood pressure assessments were conducted the day of the visit. In addition, sociodemographic data were collected. All measures were conducted by standardized trained research personnel using previously calibrated equipment.

#### Anthropometrics

Weight and height were measured following standardized procedures described by the International Society for the Advancement of Kinanthropometry (ISAK)^14^. All anthropometric measures were taken by the same previously standardized research personnel. Participants were asked to use light clothing and children were barefoot. Height was measured to the nearest 0.1 cm using a Seca 213 stadiometer (seca GmbH & Co. KG., Hamburg, Germany). Weight was measured to the nearest 0.1 kg using a calibrated digital Omron scale HBF-514C^®^ (Omron Healthcare Co, Ltd, Kyoto, Japan).

BMI z-score was calculated and classified according to the World Health Organization (WHO) age- and gender-specific growth standards with the WHO Anthro Plus software^15^. Thinness was defined as a BMI z-score < − 2 standard deviations (SD), normal weight as z-score between − 2 SD and + 1 SD, overweight as z-score >  + 1 SD, and obesity as z-score >  + 2 SD^16^.

#### Handgrip strength

It was measured using a Lafayette^®^ hand dynamometer. Patients were instructed to assume a sitting position with a straight back facing forward, with the elbow positioned at a right angle of 90° and apply as much handgrip pressure as possible by using their dominant hand. The measurements were repeated three times, and the mean value in kg of the three measurements was used for analysis. All measurements were made by a single evaluator. Children with a grip strength below the 25th percentile for age and sex were considered to have low grip strength^17^.

#### Body composition

Whole-body bioelectrical impedance analysis was performed using an RJL Systems analyzer (Quantum X, Clinton Township, MI, USA) by trained nutritionist. An alternating electric current of 800 mA at 50 kHz was applied following standard procedures^18^, and the resistance (R) and reactance (Xc) were measured.

Children were required to meet certain conditions which included fasting overnight or avoiding consumption of food and beverages for at least 4 h before the measurement. They were also not to have engaged in strenuous exercise 24 h before the measurement. Girls who were menstruating at the time of recruitment were scheduled for the study visit after menstruation ended. All children were asked to empty their bladder before and not to wear any metal object during measurement. BIA assessment was conducted at “room temperature”^19,20^.

##### Conventional BIA

R and Xc were further processed by using the Body Composition 4.0 software provided by the manufacturer. Kilograms and percent of total body weight were estimated for fat mass (FM), fat free mass (FFM), lean soft tissue (LST), and skeletal muscle mass (SMM) by using the prediction equations for pediatric population included in the software. In addition, FM and FFM indexes (FMI, FFMI) were calculated by dividing FM and FFM by height in squared to adjust for body size. Children with an FMI above the 75th percentile or FFMI below the 25th percentile for age and sex according to a reference Mexican population^21^, were considered to have high FMI or low FFMI, respectively.

##### Bioelectrical vector analysis (BIVA)

This analysis offers a semi-quantitative assessment of body cell mass and body water, and has been considered a useful method to assess hydration and nutritional status in a variety of populations at all stages of life^22^. Individual raw measurement of whole-body impedance, R and Xc, were standardized by height and were plotted on the tolerance ellipses of the Mexican pediatric population by age and sex^23^. According to the location of the quadrant outside the 75th percentile, they were classified in one of four categories: thinness (upper right), athletic (upper left), obesity (lower left) or cachexia (lower right). Children who were located within the 50th or 75th percentiles were classified as normal, regardless of the quadrant.

For group vector analysis of children according to the classification of hand-grip strength: (1) equal or greater the 25th percentile for sex and age, or (2) below the 25th percentile for sex and age, the values of R/H and Xc/H in Ohm/m were transformed into bivariate Z-scores (i.e. Z(R) and Z(Xc)) using the mean and standard deviation (SD) of the gender- and sex-specific reference healthy population of Mexican children^23^. following the methodology proposed by Piccoli et al.^24^. Mean, standard deviation and correlation coefficient of R/H and Xc/H were calculated for these two hand-grip strength groups in order to construct mean vectors for each group and identify the differences between them. Mean group vectors were represented as point vectors with their 95% confidence ellipse. Separate 95% confidence ellipses indicated a statistically significant difference between mean vector positions on the Z (R) and Z (Xc) plane.

BIVA tolerance ellipses were constructed based on data from 1331 children, 661 boys and 670 girls aged 2 to 15 years. Children were recruited from 6 schools and 5 Child Welfare Center in Mexico City in 2006^23^. Tolerance ellipses were constructed following the methodology proposed by Piccoli et al.^25^.

#### Blood pressure

Systolic and diastolic blood pressure was measured in a seating position in the right arm after a 10-min rest using an aneroid sphygmomanometer (Welch Allyn, Inc, Skaneateles Falls, New York) and a pediatric appropriately sized cuff. Three measurements were taken with 3-min intervals and the average was used for analysis. BP was classified using the population-based percentiles provided by the American Academy of Pediatrics for height, sex, and age. Elevated blood pressure was defined as systolic and/or diastolic blood pressure values ≥ 90th percentile^26^.

### Statistical analysis

Continuous variables are presented as mean ± standard deviation or median (25th, 75th percentiles), according to the variable distribution assessed by the Shapiro Wilk test. Categorical variables are presented as absolute and relative frequencies. Descriptive statistics are presented by sex. For comparison of continues variables between study groups, the Student’s t-test or the Mann–Whitney U test was employed, while comparison of categorical variables was carried out by the chi-square test or Fisher exact test.

Differences of group vectors between children with a hand-grip strength below or above the 25th percentile for sex and age was assessed by the Hotelling’s t-squared test. The receiving operating characteristic (ROC) analysis was employed to test the ability of BMI z-score, FM% and FMI to discriminate children with EBP from those with normal blood pressure through the areas under the ROC curve (AURC), for which 95% confidence intervals (CIs) were constructed. ROC curves were compared among the three adiposity indicators. All analyses were performed with commercially available software (SPSS 24.0 for Windows, SPSS, Inc, Chicago, IL), except for the bioelectrical vector analysis, for which the BIVA tolerance and confidence software were used^27^ (BIVA software is available at http://www.renalgate.it/formule_calcolatori/bioimpedenza.htm). No missing data were identified.

## Results

Overall, 180 children were assessed for eligibility and 61 agreed to participated by giving written informed consent. Those were the children included in this study. Of them, 52.5% were boys, and the median age was 9.8 (25th, 75th percentiles: 8.5, 11.0) years. Characteristics of the study sample are reported in Table 1. From all children, 49.2% had overweight or obesity. None of the studied children were classified as thin.

**Table 1:** Descriptive characteristics of the study sample by sex.

Characteristic	Overall	Boys (n = 32)	Girls (n = 29)
Age (years)	9.8 (8.5, 11.0)	9.9 (8.2, 11.0)	9.6 (9.0, 11)
Weight (kg)	34.1 (28.4, 47.3)	34.5 (28.1, 48.7)	33.9 (28.4, 47.3)
Height (cm)	139.1 ± 10.4	137.7 ± 10.4	140.6 ± 10.3
BMI (kg/m^2^)	17.9 (15.7, 23.1)	18.3 (15.9, 23.7)	17.4 (15.4, 22.2)
Weight status by Z-BMI			
Normal (n, [%])	31 (50.8)	14 (43.8)	17 (58.6)
Overweight (n, [%])	13 (21.3)	7 (21.9)	6 (20.7)
Obesity (n, [%])	17 (27.9)	11 (34.4)	6 (20.7)
Hand-grip strength (kg)	13.9 ± 4.7	14.8 ± 4.3	12.8 ± 5.1
Systolic blood pressure (mmHg)	97.3 ± 9.7	97.4 ± 10.2	97.1 ± 9.4
Diastolic blood pressure (mmHg)	61.8 ± 7.8	62.1 ± 8.5	61.4 ± 7.0

Data are reported as mean ± standard deviation or median (25th, 75th percentiles).

### Association between weight status by Z-BMI and body composition parameters

#### Weight status and conventional BIA

**Figure 1:**
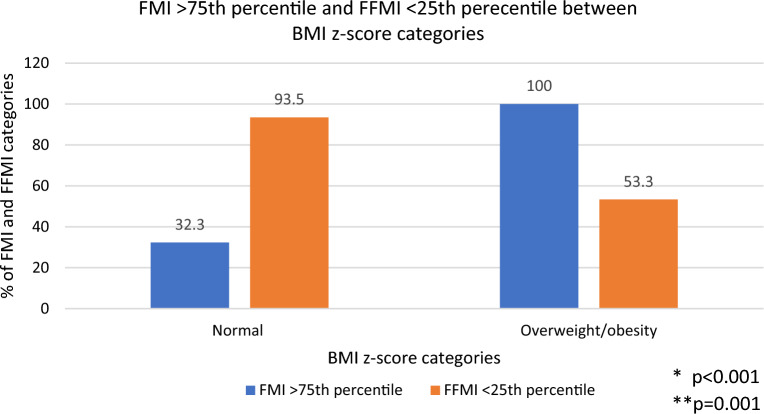
Distribution of FMI > 75th percentile and FFMI < 25th percentile between Z-BMI weight status categories in school-age children. *p value by Chi^2^ test for comparison of FMI categories between BMI z-score categories. **p value by Chi^2^ test for comparison of FFMI categories between BMI z-score categories.

**Figure 2:**
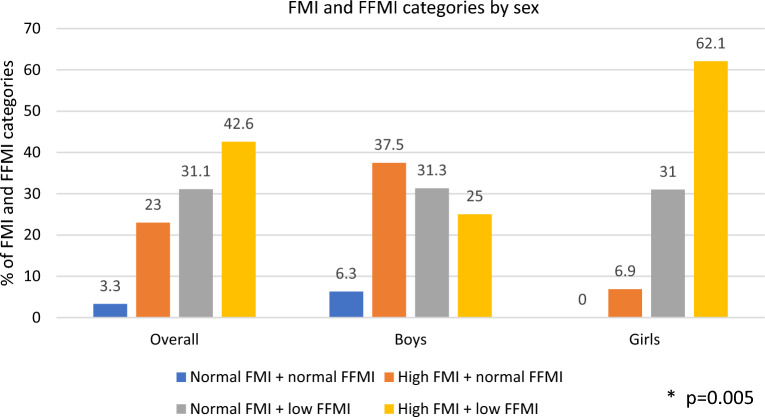
Distribution of FMI and FFMI categories in school-age children by sex. *p value by Chi2 test for comparison of categories between boys and girls.

Conventional BIA parameters for body composition assessment were different in the overall comparison among groups (Table 2). From all children, 40 (65.6%) had an FMI above 75th percentile and 45 (73.8%) displayed a FFMI below 25th percentile for age and sex (data not shown). Proportion of children with these conditions in each Z-BMI category is shown in Fig. 1. FMI > 75th percentile was observed in 32.3% of children with normal weight by Z-BMI, while all the children with overweight or obesity exhibited an MFI above 75th percentile. More than 50% of children in each Z-BMI category showed an FFMI below 25th, with a 93.5% of children having this condition among children with normal weight. Importantly, from all children, 26 (42.6%) presented both conditions high FMI and low FFMI, with a higher proportion of girls having this body composition characteristic compared to boys (62.1% vs. 25%) (Fig. 2).

**Table 2:** Body composition parameters by Z-BMI groups.

Characteristic	Normal (n = 31)	Overweight/obesity (n = 30)	Overall p value^a^
BMI z-score (SD)	− 0.5 (0.8, 0.0)	2.2 (1.3, 2.8)	< 0.001
Hand-grip strength (kg)	12.4 ± 4.7	15.4 ± 4.3	0.013
FM (kg)	7.5 ± 2.8	21.3 ± 7.6	< 0.001
FM (%)	25.1 ± 6.3	43.6 ± 7.4	< 0.001
FMI (kg/m^2^)	4.0 ± 1.2	10.4 ± 3.4	< 0.001
FFM (kg)	21.9 ± 3.8	26.4 ± 4.6	< 0.001
FFM (%)	74.9 ± 6.3	56.5 ± 7.4	< 0.001
FFMI (kg/m^2^)	11.8 ± 0.8	12.9 ± 1.0	< 0.001
LST (kg)	20.4 ± 3.6	24.7 ± 4.3	< 0.001
LST (%)	69.9 ± 6.0	52.7 ± 6.9	< 0.001
SMM (kg)	8.6 ± 1.6	11.0 ± 2.3	< 0.001
SMM (%)	29.5 ± 3.2	23.5 ± 3.2	< 0.001

Data are reported as mean ± standard deviation or median (25th, 75th percentiles).

^a^By Student’s t-test or Mann–Whitney U test for comparison between groups.

#### Weight status and bioelectrical impedance vector analysis (BIVA)

**Figure 3:**
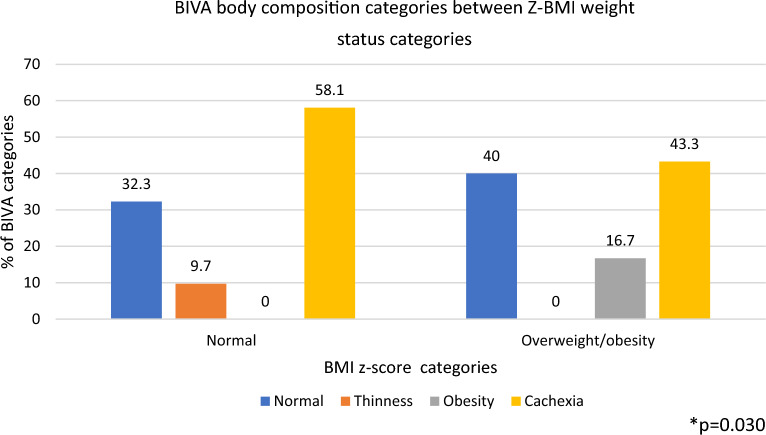
Distribution of BIVA body composition categories between BMI z-score weight status categories in school-age children. *p value by Chi2 test for comparison of BIVA categories between BMI z-score categories.

**Figure 4:**
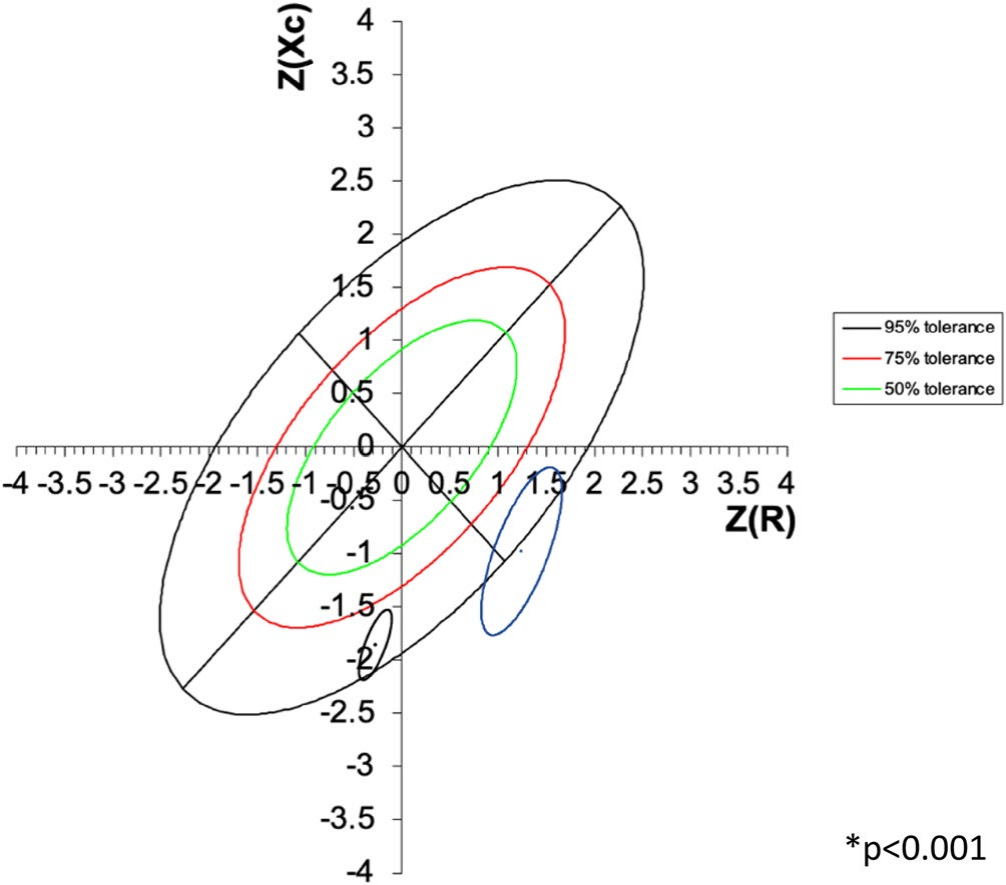
RXc z score graph showing group impedance vectors for hand-grip strength split at 25th percentile for age and sex: ≥ 25th percentile in black and < 25th percentile blue. *p value for comparing mean group vectors by the Hotelling´s t-squared test.

When weight status by Z-BMI and body composition categories by BIVA method were compared, a high proportion of participants with cachexia by BIVA was found in both Z-BMI categories, while 32.3% and 40% of children in the normal weight and overweight/obesity categories were classified as having normal body composition by the BIVA method. All children classified as having obesity by BIVA were classified as having overweight or obesity by Z-BMI, representing only the 16.7% of all children in this overweight/obesity Z-BMI category (P = 0.030) Fig. 3. Moreover, BIVA method showed that children with a hand-grip strength below the 25th percentile for age and sex exhibited a more altered body composition as denoted by a group vector located in the cachexia and thinness quadrants outside the 75th and 95th tolerance ellipses, compared to children with a hand-grip strength > 25th percentile, whose vector was in the cachexia quadrant within the 75th and 95th tolerance ellipses (Fig. 4).

### Weight status, body composition and elevated blood pressure

Frequency of elevated blood pressure (EBP) among Z-BMI and FMI-FFMI categories are shown in Table 3. Neither Z-BMI nor FMI-FFMI categories were significantly associated with EBP; however, when using FMI-FFMI categories, none of the children with normal FMI and normal FFMI were classified as having EBP. Numerically, higher proportions of EBP were seen in the categories of elevated FMI (> 75th percentile) with or without low FFMI (< 25th percentile), although this association was not statistically significant.

**Table 3:** Association between Z-BMI and FMI-FFMI categories with EBP.

BMI z-score categories	EBP (n [%])
Normal (n = 31)	1 (3.2)
Overweight/obesity (n = 30)	6 (20)
*p value among categories	0.053
FMI-FFMI categories	
Normal FMI + normal FFMI (n = 2)	0 (0)
High FMI + normal FFMI (n = 14)	2 (14.3)
Normal FMI + low FFMI (n = 19)	1 (5.3)
High FMI + low FFMI (n = 26)	4 (15.4)
**p value among categories	0.687

*By the Fisher exact test, ** By chi-square test.

Table 4 shows the areas under the ROC curves with 95% CI for Z-BMI and body composition parameters for EBP. All AURC were statistically significant, meaning that all of these parameters were able to distinguish children with EBP. Fat mass (%) showed the largest AURC for EBP (0.794); however, it was not statistically different from Z-BMI AURC (0.770) or FMI (kg/m^2^) AURC (0.775).

**Table 4:** Areas under the ROC curves for Z-BMI and body composition parameters for discriminating EBP.

	BMI z-score	FM (%)	FMI (kg/m^2^)
	AURC (95% CI) p value	AURC (95% CI) p value	AURC (95% CI) p value
EBP (≥ 90 percentile*)	0.770 (0.577, 0.962) 0.006	0.794 (0.618, 0.969) 0.001	0.775 (0.604, 0.946) 0.002

*EBP* elevated blood pressure.

*According to height, sex, and age.

## Discussion

In this study in school-age children, the main findings are as follows: First, a third of children with normal weight by BMI z-score had high adiposity (FMI > 75th percentile) and more than 90% of them were classified has having low FFMI (FFMI < 25th percentile). In addition, more than 50% of children with overweight or obesity by BMI z-score presented a low FFMI (< 25th percentile for sex and age). These data confirm that low FFMI is common in this sample of children and identifying deficiency of FFM might be hindered by using only BMI indicators. Second, BIVA method was able to identify a high prevalence of cachexia in both BMI z-score groups. The prevalence of sarcopenic obesity and its health implications in pediatric population has not been well studied due to the lack of consensus of clear diagnostic criteria in this population^11^; however, these results show that by assessing body composition by BIVA, we can identify certain phenotypes of deficiency of muscle mass and preserved or excess body fat, regardless body weight. Finally, 4 out of 7 children with EBP had a body composition phenotype characterized by high FMI and low FFMI. Whether or not this body composition phenotype implies a greater cardiometabolic risk even in children needs to be further determined in larger studies. Nonetheless, these results provide relevant information to consider the BIA methods as useful to identify different body composition phenotypes with potential health implications.

Body mass index is a good screening measure for adiposity; however, it is well known that its correlation with FMI is better at higher than lower BMI values^28–30^, and thus, adiposity may be underestimated in children with normal weight, nonetheless, weight outcomes are still the main goal for obesity managing and its related comorbidity risk^31^. Herein, we reported a greater mean FMI among children with overweight or obesity compared to their normal weight counterparts (10.4 ± 3.4 vs. 4.0 ± 1.2, < 0.001); however, when children were classified using the sex- and age-specific FMI normative values for pediatric Mexican population, more than 30% of children in the normal weight group were classified as having high FMI. These results are in congruence with those reported by Clasey et al.^7^, who reported a large proportion of children with high percent of FM (determined by BIA) among healthy weight children (defined by a BMI percentile > 5th, < 85th), particularly among boys (56/289, 19.4%), questioning the “low-risk” connotation attributed to a body weight in normal range. This is supported by the study conducted by Bai et al. in Chinese children, where it was observed a positive association between percent of FM and abnormal blood pressure in normal-weight children^6^.

Importantly, low FFMI was a frequent condition among both children with normal weight (93.5%) and overweigh/obesity (53.3%) in this study. Clasey et al.^7^ also reported a high prevalence of low FFMI among boys (49.8%) and girls (40.1%) with normal weight. Of note, more than 50% of children with overweight/obesity in our study had low FFMI, and 42.6% had both high FMI and low FFMI, suggesting a potential risk of sarcopenic obesity. Sack et al.^32^ reported a higher prevalence of sarcopenia (69.7%) as defined by using the muscle-to-fat-ratio determined by BIA, among German children and adolescents with obesity. In such study, sarcopenia was associated with higher gamma-glutamyl transferase, glutamate pyruvate transaminase, high-sensitivity C-reactive protein, and diastolic blood pressure, and lower muscular and cardiorespiratory fitness compared with obesity without sarcopenia. Despite the potential clinical consequences of sarcopenic obesity in children, a recent systematic review^11^ points out the need for population-, age-, and sex-specific definition criteria for sarcopenic obesity, as well as consensus on the valid technics for its assessment.

In our study, we reported that 6 out of 7 cases of EBP were observed among children with high FMI, with or without low FFMI, as measured by BIA. When BMI z-score, percent of FM, and FMI discriminating performance for EBP was tested, all of these adiposity indictors showed a good AURC (ranging between 0.770 and 0.794); with the highest value for percent of FM (0.794), although there were no significant differences among them. Chen et al.^33^ reported that that percent of FM was associated with EBP (adjusted odds ratio (OR): 1.043, 95%CI = 1.027–1.059) in Chinese children; however, the role of FFM or muscle mass in this association was not investigated. In our study, 4 out of 7 children with EBP had high FMI and low FFMI.

We explored the usefulness of an alternative BIVA method for body composition assessment in children. This method identified a large proportion of children presenting a body composition alteration characterized by a low content of muscle mass and preserved or increased fat mass, named as cachexia, in both the normal (58.1%) and overweight/obesity (43.3%) BMI z-score groups. In a study of 8-year-old Italian children^34^, 7 out of 218 vectors of children with normal-weight and 3 out of 135 of vectors of children with overweight by BMI fell out of the 75% tolerance ellipses of the cachexia quadrant (right and lower quadrant), while 81 out of 111 children with obesity by BMI fell within the 75% tolerance ellipse, indicating no altered body composition by BIVA. On the other hand, Oliveira et al.^35^, in a study of 78 Brazilian adolescents with obesity, reported the presence of cachexia by BIVA in 21.9% of girls and 6.5% of boys. Although these studies observed a lower proportion of cachexia compared to our findings, results are consistent in showing that weight status assessment by BMI may not identify children with an altered muscle mass phenotype. Similar findings have been observed in adult patient population studies^36,37^.

It is important to note that our study was conducted post COVID-19 pandemic, which may have influenced body composition phenotype in these children due to less physical activity associated to the long-term implemented lockdown. This may explain, at least in part, the high proportion of children with cachexia by BIVA in this study.

The BIVA method was also able to discriminate between children with low or normal grip strength for sex and age, displaying a group vector in the quadrants of cachexia and thinness outside the 75th and 95th tolerance ellipses for those with low grip strength. Children with normal grip strength exhibited shorter vectors located only in the cachexia quadrant within the 75th and 95th tolerance ellipses. A study in adult population also reported a correlation between BIVA parameters and grip strength, where Xc/H (cell membrane surface and membrane integrity indicator) was associated positively with a 0.573 kg hand grip strength increase per additional unit, while R/H (fluid volume indicator) was associated with a 0.063 kg strength decrease per unit increase^38^. Whether or not BIVA phenotypes are associated with other health outcomes in pediatric population needs to be further investigated.

Taking all together, we can highlight the relevance of body composition assessment for growth and nutritional status monitoring beyond BMI^39^, but also for identifying health risks associated with body composition alterations that guide early prevention strategies in this population, such as programs to prevent and manage excessive adiposity and deficiency of muscle mass, regardless body weight. However, it is important to taking into consideration that a proper interpretation of body composition parameters requires of normative values that are population-, sex- and age-specific, which are not always available for all populations.

This study has the following main limitations: first, the small sample size involved limited may have increased the probability of occurrence of type II error for the association between the different body composition phenotypes (identified by conventional and vectorial BIA) and EBP. However, this is the first study assessing the utility of BIVA and its association with grip-strength, an indicator of muscle function, in healthy children. Second, association between body composition and EBP needs to be considered in the context of other potential confounding variables such as physical activity and diet. However, it is important to note that BMI z-score, FMI, and FFMI were classified according to sex- and age-specific normative values, and EPB was defined according to height-, sex- and age-specific cut-offs. Third, reference population from which BIVA tolerance ellipses were constructed was limited to a modest sample of children from Mexico City; further research in larger populations is needed to develop representative normative values for BIVA. Forth, due to the nature of this study conducted outside the clinical setting, EBP was determined based on 3 blood pressure measurement taken the same day. Finally, sampling strategy of this small sample size was not probabilistic, thus, result should not be generalized to all school-age children from Mexico City and conurbation area.

## Conclusion

In this study in school-age children, low FFMI by conventional BIA and cachexia defined by BIVA were common in both children with normal weight and those with overweight or obesity; thus, identifying deficiency of FFM might be limited by using solely BMI indicators. Low grip strength was associated with a more compromised body composition by BIVA. This body composition method appears to be a useful alternative to assess body composition in children without making any assumptions about body weight.

Both BMI z-score and FM (% and FMI), performed well at discriminating EBP. The impact of low FFMI in addition to high FM on the risk of EBP and other cardiometabolic risk markers needs to be further studied in a larger population.

## Data Availability

The data that support the findings of this study are not openly available due to reasons of sensitivity and are available from the corresponding author upon reasonable request.

## Abbreviations

EBP: Elevated blood pressure
FM: Fat mass
BMI: Body mass index
LST: Lean soft tissue
DXA: Dual x-ray absorptiometry
BIA: Bioelectrical impedance analysis
BIVA: Bioelectrical impedance vector analysis
ISAK: International Society for the Advancement of Kinanthropometry
WHO: World Health Organization
R: Resistance
Xc: Reactance
FFM: Fat free mass
SMM: Skeletal muscle mass
FMI: Fat mass index
FFMI: Fat free mass index
ROC: Receiving operating characteristic
AURC: Area under the ROC curve
